# Quantum Dots for Biomedical Biosensing, NIR‐II Bioimaging, and Phototherapy: Materials Design, Signal Transduction, and Translational Barriers

**DOI:** 10.1002/advs.75491

**Published:** 2026-04-30

**Authors:** Jie Ju, Zhifang Liu, Xinran Gao, Wen Sun, Wei Fu, Yanfei Wang

**Affiliations:** ^1^ Key Laboratory of Carcinogenesis and Translational Research (Ministry of Education) Day Oncology Unit Peking University Cancer Hospital & Institute Beijing China; ^2^ Institute of Atomic Manufacturing Beihang University Beijing China; ^3^ Department of Respiratory and Critical Care Medicine Peking University First Hospital Beijing China; ^4^ Anhui Province Key Laboratory of Conservation and Utilization for Dabie Mountain Special Bio‐Resources West Anhui University Lu'an Anhui China

**Keywords:** bioimaging, biomedical applications, biosensing, cancer treatments, quantum dots

## Abstract

Quantum dots (QDs) have emerged as standout candidates among inorganic nanomaterials, distinguished by their tunable photoluminescence, exceptional photostability, and size‐dependent quantum confinement effects that enable tailored emission from the visible to the near‐infrared range. These remarkable optical properties, coupled with broad absorption spectra and high quantum yields, have positioned QDs at the forefront of diverse biomedical applications. This review provides a systematic overview of QDs fabrication strategies, with a focus on bottom‐up approaches, such as colloidal synthesis, hydrothermal, and solvothermal methods, as well as emerging biomimetic synthesis inspired by natural biomineralization. Additionally, we offer an in‐depth discussion of cutting‐edge QDs applications across three key areas: high‐sensitivity biosensing for biomarker detection and point‐of‐care diagnostics; bioimaging, including fluorescence, magnetic resonance, and photoacoustic imaging; and intelligent nanocarrier‐based cancer therapeutics, encompassing targeted drug delivery and imaging‐guided precision surgery. Furthermore, this review examines the key challenges and optimization strategies for QDs in biomedical applications, with a particular focus on the critical bottlenecks impeding their clinical translation. By analyzing these barriers and outlining future directions, it aims to provide both theoretical and practical guidance for translating QDs from laboratory‐scale innovations into routine clinical practice.

## Graphical abstract

This review outlines bottom‐up and biomimetic fabrication strategies of quantum dots, and highlights their emerging applications in biosensing, multimodal bioimaging, and intelligent cancer theranostics. It further discusses key translational barriers and future perspectives for advancing QD‐based nanomedicine toward clinical implementation.

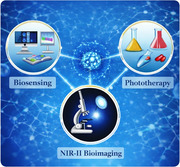

## 1 Introduction

Over the past few decades, significant strides have been made in the realm of nanomaterials. Among various inorganic nanoparticles, quantum dots (QDs) have emerged as standout candidates thanks to their remarkable tunable photoluminescence properties and exceptional resistance to photobleaching [1, 2]. QDs, characterized as monodisperse nanocrystals with exceedingly small sizes akin to the bulk‐exciton Bohr radius, exhibit a quantum confinement effect, resulting in size‐tailored emission spectra spanning from the visible to near‐infrared (NIR) regions [3, 4, 5]. Alongside this unique attribute, QDs boast a plethora of advantageous properties, including broad absorption spectra, substantial Stokes shifts, and high photoluminescence quantum yields (PLQY). These outstanding optical characteristics have propelled QDs into the spotlight across diverse applications, particularly in the biomedical arena, where their potential is extensively explored and harnessed [6]. An array of QDs with diverse structures and chemical compositions have been synthesized and investigated. These encompass perovskite QDs (PQDs) [7, 8, 9], carbon dots (CDs) [10, 11, 12], InP QDs, [13, 14], and metal chalcogenide QDs (MCQDs) [15, 16, 17], among others.

The fabrication strategies for QDs primarily encompass two paradigms: top‐down and bottom‐up approaches. The former includes physical processing techniques such as lithography and ultrasonic exfoliation, which are well‐suited for the precise construction of specific micro/nanostructures. The latter, centered on chemical synthesis, has become the mainstream route for the controllable preparation of QDs due to its precise regulation of reaction conditions and component ratios, garnering widespread favor in both academia and industry. Among the various branches of bottom‐up methods, colloidal synthesis has attracted particular attention for its high tunability over the size, morphology, and surface states of QDs [18], offering a reliable pathway for the batch production of high‐quality monodisperse QDs. Hydrothermal and solvothermal synthesis represent alternative routes that leverage high‐pressure conditions within closed systems to enable low‐temperature, greener preparation of QDs, featuring advantages such as low energy consumption, operational safety, and simple equipment [17]. Notably, biomimetic synthesis, inspired by natural biomineralization processes, is rapidly gaining ground as a sustainable and environmentally friendly paradigm [19]. By utilizing biological templates or biomolecules for directed synthesis and surface regulation, this strategy not only substantially curtails the use of toxic precursors and the generation of hazardous waste but also imparts exceptional intrinsic biocompatibility to QDs. In doing so, it helps overcome key obstacles hindering their direct deployment in biomedical applications. Thus, from precisely controllable colloidal chemistry to environmentally friendly biomimetic strategies, the continuous innovation and high adaptability of QDs synthesis technologies are steadily pushing the boundaries of their performance. These advances lay a solid material foundation for the realization of diverse application scenarios, including high‐performance biosensors, bioimaging, and targeted theranostics.

In this review, we first discuss how bottom‐up and biomimetic synthesis strategies determine the size, composition, surface chemistry, and optical properties of QDs. We then examine how these features govern signal generation and biological performance in biosensing, including biomarker detection, rapid virus detection, and point‐of‐care testing (POCT), as well as in bioimaging, such as fluorescence imaging, magnetic resonance imaging (MRI), and photoacoustic imaging (PAI), and in cancer therapy. Finally, we discuss how the same material characteristics that enable biomedical function can also create challenges for clinical translation, particularly in biosafety, in vivo behavior, reproducibility, and scalable manufacturing. In this way, the review connects QD design with biomedical application and translational feasibility.

## 2 Fabrication of QDs

Given the remarkable advantages of bottom‐up synthesis strategies in precise composition control, uniform size and morphology regulation, as well as surface functionalization modification, they have emerged as the mainstream paradigm for the controllable preparation of QDs [20, 21]. Accordingly, this review focuses on an in‐depth discussion of this strategic framework. Specifically, it covers three representative methods: colloidal synthesis; hydrothermal/solvothermal synthesis; and biomimetic synthesis.

The colloidal synthesis method, first systematically established by Murray, Norris, and Bawendi in 1993 [22] enables atomic‐level control over the size, composition, and morphology of QDs through precise regulation of key parameters such as reaction temperature, duration, precursor type and ratio, ligand species, and concentration. Since its inception, this method has undergone continuous optimization, gradually evolving into a variety of technical variants, including high‐temperature organic‐phase synthesis, aqueous‐phase synthesis, and continuous‐flow reaction systems. Among these, the high‐temperature organic‐phase route remains the preferred strategy for preparing high‐performance QDs, owing to its advantages in controllable reaction kinetics, high crystallinity of products, and low surface defect density [23]. This approach offers exceptional flexibility and scalability: by adjusting reaction conditions, QDs with tunable sizes (typically 2–20 nm), morphologies (spherical, rod‐shaped, core–shell structures, etc.), and compositions (binary, ternary, gradient alloy, etc.) can be reproducibly synthesized. Such versatility endows the method with remarkable design freedom and broad application adaptability [24]. The precise controllability inherent to colloidal synthesis not only provides an ideal model system for investigating the quantum confinement effect and structure‐property relationships, but also establishes an indispensable methodological foundation for the targeted optimization of QDs performance, such as near‐unity PLQY and single‐photon emission, as well as multifunctional integration, including core–shell engineering and heterojunction design. As such, it serves as a critical technological pillar enabling the translation of QDs from fundamental research into practical device applications.

The hydrothermal/solvothermal method relies on a high‐temperature and high‐pressure environment within a sealed autoclave, using water or organic solvents as reaction media. Through external heating, the precursor solution containing raw materials and ligands reaches a supersaturated state, thereby inducing homogeneous nucleation and controlled growth of QDs. This approach leverages the significant increase in solvent vapor pressure with rising temperature in a confined system, enabling reaction kinetic activation at temperatures far below the decomposition point of precursors, effectively promoting crystal nucleation and oriented crystallographic growth. Compared with high‐temperature organic‐phase synthesis, the hydrothermal/solvothermal method offers several distinct advantages. First, it is straightforward and cost‐effective, requiring neither inert gas protection nor complex high‐vacuum systems, which substantially reduces equipment investment and operational barriers. Second, it features mild reaction conditions, typically completed within 100°C–250°C, leading to lower energy consumption and significantly enhanced operational safety, making it particularly suitable for integrating thermally sensitive functional components. Third, it provides rich regulatory dimensions: by systematically adjusting parameters such as reaction temperature, autoclave pressure, reaction time, solution pH, and the type of mineralizer, fine control over the size distribution, morphological features, crystal phase structure, and surface chemistry of QDs can be achieved [25, 26]. Fourth, it offers green chemistry potential, using water or low‐toxicity alcohols as solvents in alignment with sustainable development principles, thus providing a viable pathway for the large‐scale green production of QDs. This method has proven especially effective in the preparation of CDs, silicon QDs, and metal chalcogenide QDs. The resulting products often exhibit excellent water solubility and intrinsic biocompatibility, enabling their direct application in biolabeling and medical diagnostics. Moreover, the hydrothermal/solvothermal environment provides unique thermodynamic conditions for in situ doping, surface functionalization, and heterostructure construction, facilitating the formation of metastable phases or complex compositional ratios that are difficult to achieve via conventional methods. Thus, it opens important experimental avenues for the exploration of novel quantum dot material systems.

Biomimetic synthesis, inspired by natural biomineralization processes, leverages the inherent molecular recognition, templating effects, and catalytic activities of biological systems to direct the controlled nucleation and growth of QDs under mild conditions. This method employs enzymes, proteins, peptides, nucleic acids, microorganisms (such as bacteria, fungi, and algae), or their metabolites as biological templates and reaction media. Through the specific coordination interactions between biomacromolecules and metal ions, precise control over the size, morphology, crystal phase, and surface chemistry of QDs is achieved. The core mechanism involves the synergistic interplay of spatial confinement effects from biological templates, functional group‐induced heterogeneous nucleation, and biochemical reactions such as bioreduction or biosulfurization. This approach offers multiple advantages that are difficult to realize with conventional chemical synthesis. First, the reaction conditions are exceptionally mild, typically carried out at ambient temperature and pressure, near‐neutral pH, and in aqueous environments, completely avoiding the flammable and explosive reagents and stringent operating conditions associated with high‐temperature organic‐phase synthesis. Second, it demonstrates broad material adaptability, having been successfully extended to a wide range of quantum dot systems, including II‐VI (CdS, ZnSe), IV‐VI (PbS), I‐VI (Ag_2_S), and carbon‐based QDs, indicating extensive synthetic versatility [27]. Third, it exhibits outstanding green and sustainable characteristics, significantly reducing the use of toxic precursors, organic solvents, and strong reducing agents, thereby minimizing environmental burden and carbon footprint at the source. Fourth, it endows QDs with excellent intrinsic biocompatibility: biological template molecules can be directly anchored onto the QDs surface, forming stable bio‐inorganic hybrid interfaces that impart good water dispersibility, colloidal stability, and low cytotoxicity—enabling direct use in biomedical applications without complex post‐synthetic surface modifications [28, 29]. This methodology not only deepens the understanding of bio‐inorganic interfacial interaction mechanisms but also serves as a compelling paradigm for the “learning from nature” philosophy in materials design. Moreover, through genetic engineering of biological templates or in vitro reconstruction of mineralization‐active sites, the controllability and functional diversity of biomimetic synthesis can be further expanded. These advances are expected to drive the evolution of next‐generation QDs technologies toward greener, smarter, and more life‐compatible systems.

Taken together, advances in QD synthesis not only enable precise control over structural and optical properties, but also directly dictate their functional performance in biomedical systems. In the following sections, we focus on how these material properties are translated into signal generation, biological interaction, and therapeutic efficacy across biosensing, imaging, and treatment applications.

## 3 Biomedical Applications of QDs

### 3.1 Biosensing

Biosensing is a rapidly evolving field that leverages the interaction between biological molecules and materials to detect and quantify various substances at the molecular level. The development of biosensors has been significantly enhanced by the incorporation of QDs, which are semiconductor nanocrystals with distinct electronic and optical properties. These properties make QDs highly suitable for a wide range of biosensing applications, including the detection of biomarkers, tracking of diseases, and monitoring of cellular processes. The use of QDs in biosensing has opened new avenues for improving diagnostic accuracy, sensitivity, and the real‐time analysis of biological systems. QDs are increasingly used in biosensing due to their unique optical properties, which are ideal for detecting biomarkers and tracking viral infections. For instance, Si‐CdTe QDs serve as sensitive fluorescent probes for detecting hydrogen peroxide and glucose, which are crucial for monitoring oxidative stress in diseases. These probes offer low toxicity, high specificity, and are effective in reducing biological interference, which enhances their practical application in live‐cell imaging. Furthermore, QDs facilitate the monitoring of epithelial‐mesenchymal transition (EMT), a key process in cancer metastasis, by detecting specific biomarkers like E‐cadherin with high precision. This capability is crucial for early cancer diagnosis and understanding tumor progression. In virology, QDs enhance single‐virus tracking (SVT), allowing detailed observation of viral behaviors and interactions within cells over time. This is vital for studying infection mechanisms and testing antiviral drugs. Additionally, advanced QDs‐based probes have been developed for dynamic imaging of viral infections such as viral encephalitis, which is capable of crossing the blood‐brain barrier and providing real‐time monitoring of disease progression. These applications highlight the significant role of QDs in advancing diagnostic technologies and medical research.

#### 3.1.1 Detection of Biomarkers

**FIGURE 1:**
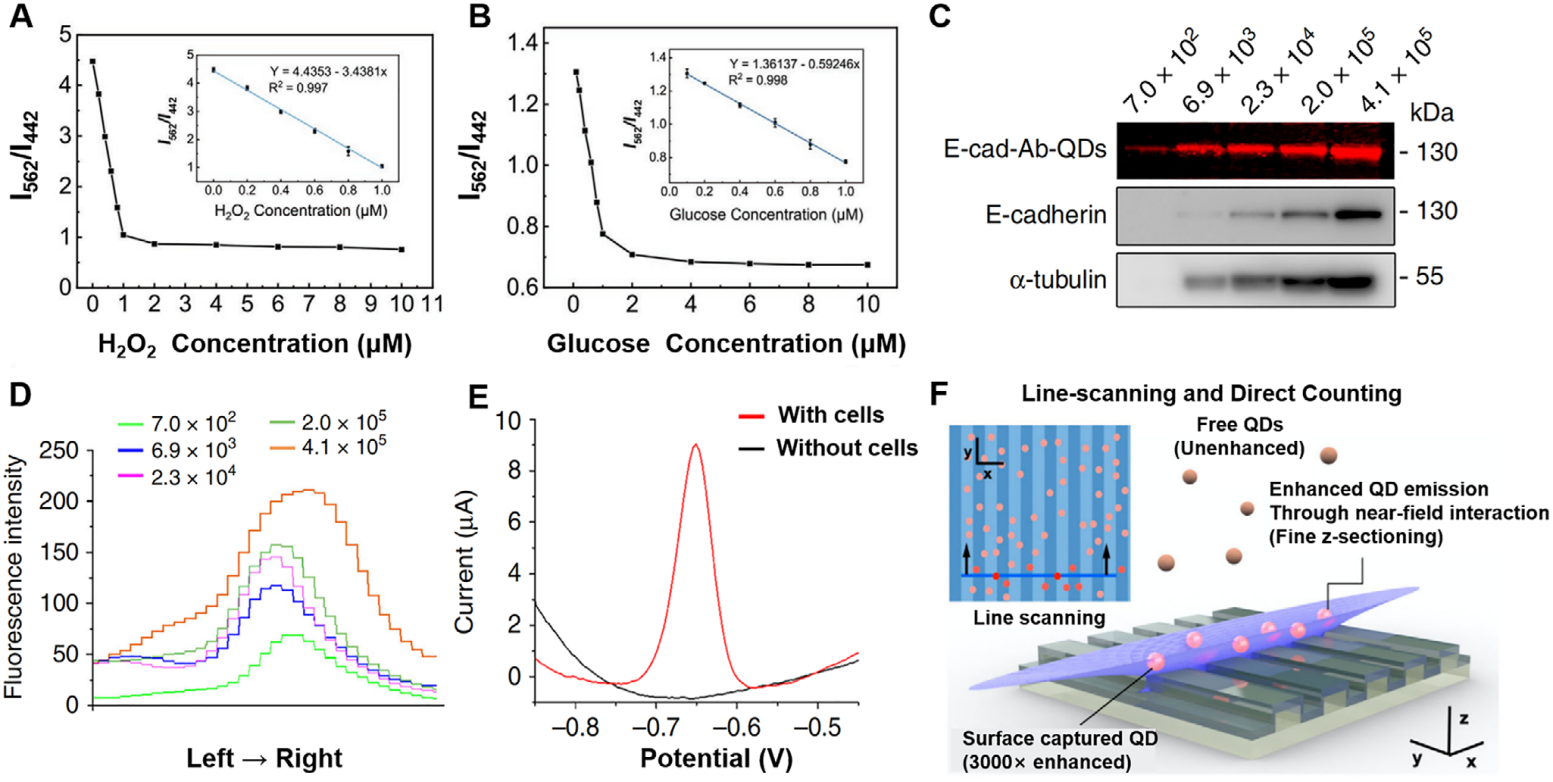
Linear plots depict the luminescence intensity ratio (I_442_/I_562_) of the Si‐CdTe QDs probe as a function of (A) H_2_O_2_ concentration and (B) glucose concentration. Reproduced with permission [32]. Copyright 2022, Wiley‐VCH. (C) Immunoblotting of E‐cadherin with the E‐cad‐Ab‐QD probe and standard E‐cadherin antibodies utilizing varying amounts of A549 cells. (D) Fluorescence intensity curves of E‐cadherin obtained from different cell quantities. (E) Response currents of the electrochemical biosensor measured with or without A549 cells via DPV. Reproduced with permission [37]. Copyright 2020, Springer Nature. (F) Dose‐response curve illustrating various concentrations across a 109‐fold concentration range after a 2‐h incubation for digital counting results. Error bars denote the mean and standard deviation of n = 3 independent assays. Reproduced with permission [38]. Copyright 2022, Springer Nature.

Reactive oxygen species (ROS) are known for their high reactivity with a wide range of biological entities [30]. Considered the most potent free radicals, they wield significant influence over diverse physiological processes like cell signaling and aging. As a pivotal ROS, hydrogen peroxide (H_2_O_2_) serves as a crucial indicator of oxidative stress in specific diseases [31]. Yet, monitoring H_2_O_2_ encounters hurdles due to the inherent autofluorescence of biological samples. Addressing this challenge, Feng and co‐workers et al. introduces Si‐CdTe QDs as innovative ratiometric fluorescent probes adept at sensitively detecting both H_2_O_2_ and glucose [32]. The luminescence ratio I_562_/I_442_ exhibited an outstanding linear relationship with the concentrations of H_2_O_2_ and glucose. The detection range for both was 0.2–10 µm (Figure 1A,B). After calculation, the LOD of H_2_O_2_ and glucose is 79 and 140 nm, respectively. The integration of CdTe QDs with Si QDs not only enhances detection capabilities but also mitigates the cytotoxicity typically associated with CdTe. Notably, incubating HeLa cells with Si‐CdTe QDs at a concentration of 500 µg mL^−1^ for 24 h yields a commendable 80% survival rate. Utilizing fluorescence extinction across 488 and 514 nm channels as internal references ensures immunity to environmental interference while showcasing selective H_2_O_2_ biosensing. Furthermore, the probe exhibits exceptional colocalization within lysosomes under the 514 nm channel, boasting a maximum Pearson coefficient of 0.96. The Si‐CdTe QDs’ advantageous attributes, including facile preparation, low toxicity, resilience to photobleaching, and precise specificity, herald a novel and convenient approach for monitoring and discerning alterations in endogenous intracellular H_2_O_2_ levels, alongside facilitating the selection of 514 nm lysosomal colocalization dyes.

Epithelial‐mesenchymal transition (EMT) holds a pivotal role in various biological applications [33], including embryonic development, tissue morphogenesis, and wound healing. Moreover, mounting evidence underscores its significance in tumor progression, wherein EMT facilitates tumor cell infiltration into neighboring tissues and metastasis to distant sites [34]. Throughout EMT, epithelial cells undergo depolarization, lose intercellular junctions, adopt a spindle‐like morphology, and acquire enhanced motility. Several molecules, notably transforming growth factor β (TGF‐β) and epidermal growth factor, act as triggers for EMT. Additionally, molecular events such as transcription factor activation, specific protein expression, and cytoskeletal rearrangements contribute to this process. Crucially, the downregulation of E‐cadherin, a member of the cadherin family, serves as a pivotal event in initiating EMT [35]. E‐cadherin expression inversely correlates with the pathological classification and staging of various cancers, rendering it a valuable biomarker for tumor diagnosis [36]. CdSe/ZnS QDs emerge as promising candidates for constructing electrochemical biosensors targeting EMT due to their unique attributes, including high electron density and size‐dependent, tunable, and narrow fluorescence emission spectra. These features enable synchronous detection of fluorescent and electrochemical signals within cells. Liu and co‐workers et al. have devised an electrochemical biosensor for EMT, employing E‐cadherin antibody‐QD (E‐cad‐Ab‐QD) conjugates as dual optical/electrochemical labels and a carbon nanotube‐gold nanoparticle (CNT‐AuNP)‐modified electrode as the detection platform [37]. When comparing E‐cadherin obtained from varying cell quantities using E‐cad‐Ab‐QDs vs. standard E‐cadherin antibodies, notable differences emerged. Polyvinylidene difluoride membranes stained with E‐cad‐Ab‐QDs exhibited superior brightness compared to those stained with standard E‐cadherin antibodies (Figure 1C). Furthermore, fluorescence intensity correlated positively with cell quantity, as evidenced by increased brightness with higher cell amounts (Figure 1D). Electrochemical detection was conducted both in the presence and absence of A549 cells. The results demonstrated that the distinct electrochemical signal observed at −0.65 V corresponded to E‐cadherin present on the cell surface (Figure 1E). Their electrochemical sensing system demonstrates specific, rapid, and sensitive detection of E‐cadherin alterations. Furthermore, this biosensor exhibits proficiency in discerning EMT stages in cells and distinguishing circulating tumor cells from those in tumor tissues in situ. Moreover, it is capable of analyzing the EMT process across various fluid samples. These findings underscore the utility of electrochemical sensing as a convenient and sensitive technique for detecting EMT.

While nanoscale quantum emitters serve as effective markers for probing biomolecular interactions, their utility in applications requiring single‐unit observations is constrained by factors such as the necessity for large numerical aperture (NA) objectives, fluorescence intermittency, and suboptimal photon collection efficiency stemming from omnidirectional emission. Cunningham and co‐workers et al. propose a sensing approach tailored for the highly specific detection of cancer‐specific miRNA targets, achieving digital resolution of individual molecules with optically enhanced high signal‐to‐noise ratios [38]. Through quality factor engineering, they achieve a remarkable ∼3000× enhancement in detected photon intensity from individual QDs tags compared to detection on a plain, unpatterned glass surface. The complementary nature of this base‐pair structure allows the target miRNA to function as a bridge, stabilizing the connection between the capture and QD‐ssDNA probe by forming a DNA‐RNA duplex. Without the target miRNA bridge, the fluorescent tags (QD‐ssDNA probe) will not be pulled down to the PC surface and thus will not experience the aforementioned 3000× enhancement, even if illuminated by the excitation laser (Figure 1F). This enhancement is attributed to a 23× gain in excitation, a 39× gain in photon extraction (comprising improvements in photon extraction rate and quantum efficiency via the Purcell effect), and a 3.5× gain in collection efficiency, as supported by experimental characterization and electromagnetic simulations. Furthermore, the photonic crystal (PC) employed in the system suppresses blinking, increasing the QDs’ on‐time from 15% to 85%, thus mitigating signal intermittency issues and enabling rapid motion tracking at the single particle level. Leveraging these synergistic properties, the PC‐QD system achieves single QD sensitivity with a high signal‐to‐noise ratio (∼59) using a low NA lens (NA = 0.5, 50×), eliminating the need for total internal reflection fluorescence (TIRF) or high‐gain electron‐multiplying cameras. This exploration of physical principles aims to enhance excitation, extraction, emission rate, and collection efficiency from individual QDs, driven by the imperative to develop sensitive, quantitative, and user‐friendly methods for detecting cancer‐related biomarkers in small clinical samples. Additionally, the study demonstrates the utility of single‐QD imaging for a digital‐resolution biomolecular assay, facilitating sensitive and selective detection of miRNA biomarkers, with the potential for adaptation to detect other miRNAs, DNAs, and proteins. Utilizing the PC‐QD system, they implement a highly specific two‐step miRNA assay with room temperature operation from a small sample volume, achieving digital resolution of individual target molecules and a detection limit of ∼10 aM, along with single base‐pair mismatch selectivity and a wide dynamic range (9 orders of magnitude). Remarkably, the blinking suppression capability enables dynamic trajectory recording of single QDs, allowing discrimination of single base differences in target miRNA molecules within 10 min without the need for washing steps. This strategy addresses clinical requirements for simple, sensitive, quantitative, and low‐volume miRNA detection, circumventing the nonlinearities associated with multi‐step enzymatic amplification by employing single‐endpoint optical detection using QD tags and a PC surface, facilitating direct counting of miRNA molecules.

#### 3.1.2 Detection of Virus

**FIGURE 2:**
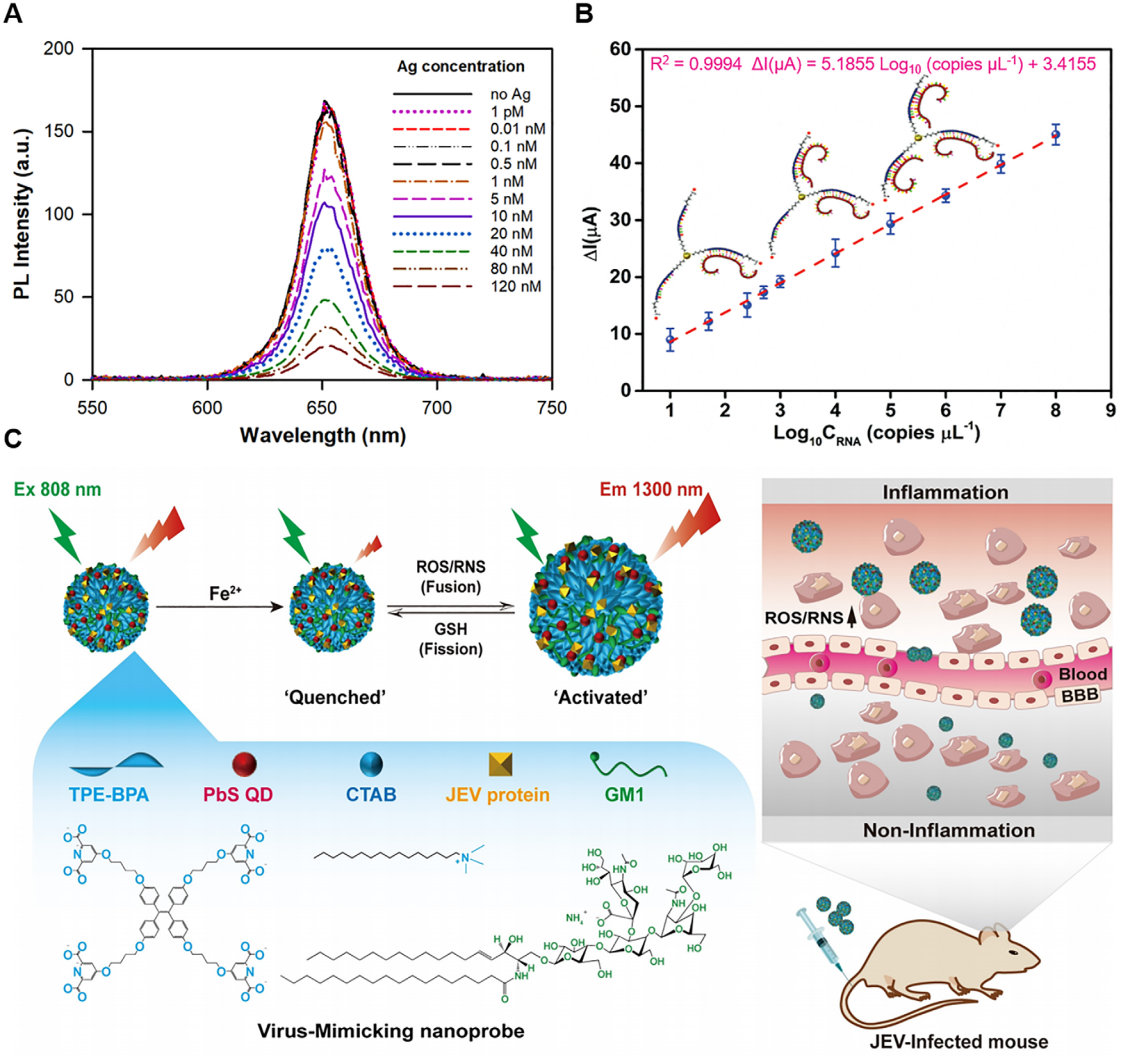
(A) PL emission spectra at different Ag concentrations from 0.001 to 120 nM. Reproduced with permission [43]. Copyright 2021, Springer Nature. (B) Linear plot of logarithmic SARS‐CoV‐2 RNA concentration vs. ΔI (µA), and the error bar indicates five independent measurements. Reproduced with permission [44]. Copyright 2023, Wiley‐VCH. (C) Schematic of the QDs&Fe^2+^@VVesicle composition and mechanism of ROS/RNS detection in vivo. Reproduced with permission [45]. Copyright 2022, Wiley‐VCH.

Single‐virus tracking (SVT) offers the opportunity to monitor the journey of individual viruses in real‐time and explore the interactions between viral and cellular structures in live cells. This exploration assists in characterizing the complex infection process and revealing associated dynamic mechanisms [39, 40]. However, conventional fluorescent tags, such as organic dyes and fluorescent proteins, suffer from low brightness and poor photostability, greatly limiting the development of SVT techniques. Challenges persist in performing multicolor SVT over extended periods. QDs present a solution with their outstanding photostability, high brightness, and narrow emission spectra tunable across a range of colors. QD‐based SVT (QSVT) allows us to track the fate of individual viruses interacting with various cellular structures at the single‐virus level, from milliseconds to hours [41, 42]. Park and co‐workers et al. have developed a highly sensitive immunosensor utilizing streptavidin‐conjugated QDs (QDs/SA) to detect the dengue biomarker non‐structural protein 1 (NS1) even at very low concentrations, enabling early‐stage detection of dengue infection [43]. Initially, the QDs/SA were bound to biotinylated NS1 antibodies (Ab), forming QDs/SA‐Ab conjugates, which were then employed to detect NS1 antigen (Ag) within the concentration range of 1 pM to 120 nM (Figure 2A). Confirmation of QDs/SA‐Ab and QDs/SA‐Ab‐Ag conjugates formation was attained through field emission scanning electron microscopy (FE‐SEM), field emission transmission electron microscopy (FE‐TEM), dynamic light scattering (DLS), and zeta‐potential measurements. Fluorescence emission spectra of QDs/SA‐Ab‐Ag conjugates demonstrated that fluorescence quenching was linearly proportional to NS1 Ag concentration, following the Stern‐Volmer (SV) equation in phosphate buffer solution. However, fluorescence quenching behavior in human plasma serum solution exhibited negative deviation from the SV equation, presumably due to interference from serum component biomolecules, elucidated by the Lehrer equation. These findings indicate the promising nature of this approach, owing to its high sensitivity, rapidity, simplicity, and convenience, thus showcasing the potential for point‐of‐care applications. Cho and co‐workers et al. present a novel approach utilizing a specially designed gold (Au)‐deoxyribonucleic acid (DNA)‐cadmium telluride (CdTe) QD probe to target two sections of the nucleocapsid (N) gene within the ribonucleic acid (RNA) of three SARS‐CoV‐2 variants (B.1.1.529, B.1.617.2, and B.1.351) [44]. Leveraging a duplex‐specific nuclease (DSN)‐assisted highly selective release of signaling probes enhances specificity, while an Au‐supported DNA probe carries multiple CdTe QD signaling probes. Upon dissolution, Cd^2+^ ions are quantified at a novel cobalt sulfide (CoS)‐nitrogen‐doped graphene QD (NGQD)/platinum (Pt)@palladium (Pd) electrode with extraordinary sensitivity via square wave anodic stripping voltammetry (SWASV). The developed sensor demonstrates a wide detection range (10 to 10^8^ copies µL^−1^, Figure 2B) and an impressively low detection limit (0.12 copies µL^−1^) without requiring amplification. Selectivity testing against MERS and HCoV‐NL63 confirms the sensor's specificity, and real‐time detection on heat‐inactivated viral samples exhibits excellent selectivity.

Viral encephalitis, an inflammatory condition affecting the brain parenchyma, stems from various viral infections. Monitoring the progression of these infections in vivo is pivotal for accurate diagnosis and timely intervention. Zhao and co‐workers et al. introduced a groundbreaking solution: an activatable and reversible virus‐mimicking near‐infrared II nanoprobes (Figure 2C) [45]. This innovative nanoprobe comprises a Fe^2+^‐coordinated, viral protein‐decorated vesicle enclosing PbS QDs emitting fluorescence at 1300 nm. Notably, the probe demonstrates the ability to traverse the blood‐brain barrier and track real‐time changes in reactive oxygen and nitrogen species concentrations during viral infection. This is achieved by modulating the quenching level of QDs and regulating vesicle fusion‐fission behavior through alterations in Fe oxidation state. Such a switching mechanism effectively mitigates background noise and enhances detection sensitivity. Consequently, this nanoprobe emerges as a promising imaging tool for dynamically visualizing viral encephalitis and holds immense potential for future clinical applications.

In conclusion, compared with traditional probes, quantum dot‐based biosensing systems exhibit superior sensitivity, multiplexing capabilities, and optical stability. However, the performance of QDs in biosensing is more influenced by the coupling between light output, surface functionalization, and the analyzed structure, rather than being affected by any single component alone. Heavy metal‐based QDs typically offer high brightness and narrow emission for ultrasensitive fluorescence readout, while carbon‐based and silicon‐based QDs are usually more attractive when low toxicity and water compatibility are given priority. On various platforms, the most successful systems tend to combine effective signal amplification with powerful anti‐fouling behavior and selective biometric recognition. However, there are still challenges in terms of reproducibility, biological interference and clinical standardization.

### 3.2 Bioimaging

Bioimaging is a fundamental aspect of modern biomedical research and clinical diagnostics, encompassing a variety of techniques that allow for the visualization of biological processes at the molecular and cellular levels. The development of advanced imaging agents and technologies has been pivotal in enhancing the resolution, sensitivity, and specificity of these imaging modalities. QDs significantly enhance bioimaging techniques across fluorescence imaging, MRI, and PAI. In fluorescence imaging, QDs improve photostability and brightness for real‐time cellular visualization, while advanced methods like FRET and QD‐SABER boost specificity and enable complex multiplexing. For MRI, QDs are used to create dual‐function probes that combine fluorescent and magnetic capabilities, enhancing imaging precision and safety through innovations in nanoparticle chemistry. In PAI, QDs enhance contrast and resolution, particularly in tumor imaging, aiding both diagnosis and therapy. These advancements underscore the critical role of QDs in pushing the boundaries of medical imaging technologies, offering deeper insights and more efficient therapeutic options in clinical settings.

#### 3.2.1 Fluorescence Imaging

**FIGURE 3:**
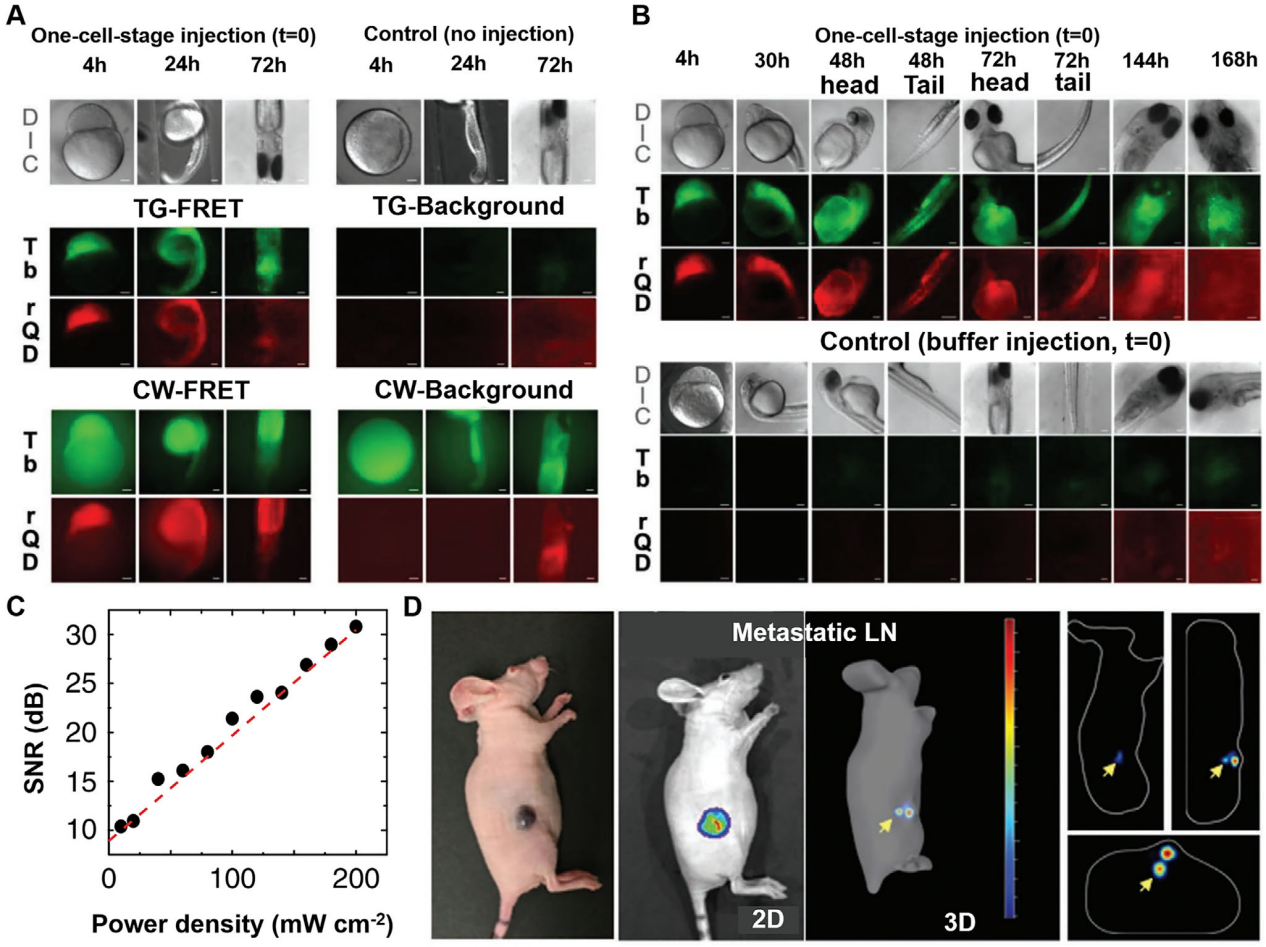
TG‐FRET imaging was conducted on developing zebrafish embryos over seven days following injection of Tb‐rQD FRET nanoprobe at the one‐cell stage. (A) Differential interference contrast (DIC), total internal reflection fluorescence (TG), and continuous wave photoluminescence (CW PL) images of zebrafish embryos at 4, 24, and 72 h post‐fertilization (hpf). (B) DIC and TG PL images of zebrafish embryos from 4 hpf to 7 days post‐fertilization (dpf). Reproduced with permission [51]. Copyright 2020, Wiley‐VCH. (C) Power density dependence of signal‐to‐noise ratio (SNR) calculated from in vivo near‐infrared II (NIR‐II) fluorescence images at various excitation power densities. Reproduced with permission [57]. Copyright 2020, Springer Nature. (D) Formation of metastatic lymph nodes following subcutaneous inoculation of 4T1 tumor. Reproduced with permission [59]. Copyright 2020, Wiley‐VCH.

Fluorescence microscopy stands as a cornerstone method for probing biological materials and their biomolecular interactions with unparalleled sensitivity at both cellular and subcellular levels, both in vitro and in vivo [46]. However, the intrinsic autofluorescence of biological components can lead to substantial background noise, particularly pronounced in tissues and living organisms. To mitigate this, a range of physical, chemical, and biological strategies have emerged, including the use of nanoparticle‐based probes that operate within the infrared spectral range [47, 48]. Beyond autofluorescence, fluorescence imaging in living organisms presents a multitude of challenges. Ideal fluorescent probes must exhibit brightness, resistance to photobleaching, minimal cytotoxicity, and compatibility with cellular biology, while enabling multiplexed subcellular molecular imaging. However, no single probe fits all needs perfectly. Fluorescent proteins (e.g., GFP), organic dyes, and nanoparticles (QDs) each possess distinct advantages and limitations, necessitating careful consideration for specific imaging studies. Luminescent semiconductor QDs offer several advantages, including high brightness, photostability, tunable emission colors, and narrow spectra. While concerns regarding their toxicity persist, surface modifications can mitigate these issues, rendering them suitable for intracellular fluorescence imaging in vitro and in vivo [49, 50]. Hildebrandt and co‐workers et al. introduce a novel terbium to QD Förster resonance energy transfer (FRET) nanoprobe characterized by exceptional brightness and photostability, coupled with narrow and adjustable emission bands, tailored for intracellular in vivo imaging [51]. Leveraging the extended photoluminescence (PL) lifetime of the probe allows for time‐gated (TG) detection, effectively eliminating the autofluorescence background. The versatility of these TG‐FRET nanoprobes is showcased through intracellular four‐color multiplexing, achieved with a single excitation wavelength, and demonstrated through in situ assembly and FRET interactions with mCherry. illustrates the schematic assembly of mCherry‐His6 (mCh) to the FRET nanoprobe, giving rise to both Tb‐to‐mCh FRET and tQD‐to‐mCh FRET interactions. Furthermore, the study underscores the potential for in vivo bioconjugation to fluorescent proteins (FPs), enabling combined nanoprobe‐FP FRET sensing. Injection of the FRET nanoprobes at the one‐cell stage permits imaging within developing zebrafish embryos for up to seven days (Figure 3A,B), exhibiting toxicity levels akin to injected RNA while significantly enhancing signal‐to‐background ratios compared to non‐TG imaging approaches. This innovative approach extends the boundaries of in vivo fluorescence imaging beyond the capabilities of conventional fluorescent proteins, presenting a promising strategy for future applications in this field. Gao and co‐workers et al. introduced a novel approach termed Quantum Dot and Signal Amplification by Exchange Reaction (QD‐SABER) for sensitive and multiplexed imaging of endogenous proteins [52]. Compared to conventional IHC processes utilizing dye‐labeled secondary antibodies, which already incorporate a signal amplification mechanism, QD‐SABER offers an additional 7.6‐fold signal amplification. Furthermore, the DNA hybridization‐based IHC can be swiftly removed, allowing sample regeneration for successive cycles of immunostaining (>10 cycles), vastly expanding multiplexing capabilities. Primary antibodies, each tagged with distinct oligonucleotide sequences (bridge oligos), are simultaneously introduced to cells. These bridge oligos act as identifiers for individual antibodies and facilitate the anchoring of orthogonal single‐stranded DNA (ssDNA) concatemers, synthesized beforehand in vitro using Polymerase End Replacement (PER). Simultaneously, fluorescent imagers for hybridization with extended concatemers are generated through a straightforward combination of biotinylated oligos with QD‐streptavidin complexes. The elusive potential of unique and pivotal bioimaging applications in vivo has been addressed by Sun and co‐workers et al., who introduced planted graphene QDs (GQDs) as an exceptional tool for in vivo fluorescent, sustainable, and multimodal tumor bioimaging across diverse scenarios [53]. Employing a bottom‐up molecular approach, GQDs are in situ planted within the poly(ethylene glycol) (PEG) layer of PEGylated nanoparticles, yielding the NPs‐GQDs‐PEG nanocomposite. These implanted GQDs exhibit over four times prolonged blood circulation and 7–8 times increased tumor accumulation compared to conventional GQDs in vivo. Following accessible specificity modification, the multifunctional NPs‐GQDs‐PEG offer targeted, multimodal molecular imaging for various tumor models, both in vitro and in vivo. Furthermore, the highly photostable nature of GQDs enables long‐term, real‐time visualization of the local pharmacokinetics of nanoparticles in vivo. This strategy of planting GQDs in PEGylated nanomedicine represents a novel approach for broadening in vivo biomedical applications of GQDs.

Fluorescence within the second near‐infrared window (NIR‐II, 900–1700 nm) has garnered significant attention for bioimaging due to its impressive tissue penetration depth and spatiotemporal resolution. There is a pressing need for NIR‐II fluorophores characterized by high PLQY, stability, and biocompatibility. Specifically, recently developed NIR‐II Ag_2_X (X = S, Se, Te) QDs have garnered attention for their exceptional optical properties and biocompatibility [54, 55]. Wang and co‐workers et al. have achieved a breakthrough by developing Ag_2_Te@Ag_2_S core–shell QDs with tunable and intensified emissions ranging from 1300 to 1560 nm through a straightforward colloidal approach [56]. They utilized enriched Ag ions on the surface of Ag_2_Te QDs as a readily available source for Ag, reacting with subsequently injected sulfur precursors to form the Ag_2_Te@Ag_2_S core–shell QDs. Notably, the Ag_2_Te@Ag_2_S QDs exhibit superior photoluminescence properties compared to Ag_2_Te QDs, showcasing significant enhancements in both photoluminescence intensity and stability. Crucially, this method enables the synthesis of core–shell QDs with various core compositions, facilitating the production of bright NIR‐IIb QDs suitable for in vivo imaging. Remarkably, Ag_2_Te@Ag_2_S QDs emitting at 1560 nm provide high spatial resolution images of deep‐seated organs and vascular structures in mice. This innovative core–shell synthetic approach holds promise for application in other narrow bandgap QDs, suggesting that core–shell NIR‐II nanoprobes have vast potential in biomedical contexts. Rubio‐Retama and co‐workers et al. introduce a pioneering advancement in probe technology with the development of Ag_2_S superdots, derived from chemically synthesized Ag_2_S dots [57]. A protective shell is meticulously grown onto these dots using femtosecond laser irradiation, effectively mitigating structural defects and yielding an impressive 80‐fold enhancement in quantum efficiency. The resultant PEGylated Ag_2_S superdots facilitate deep‐tissue in vivo imaging at remarkably low excitation intensities (<10 mW cm^−2^ Figure 3C) and doses (<0.5 mg kg^−1^), positioning them as unparalleled contrast agents for NIR‐II preclinical bioimaging. These findings underscore a novel strategy for crafting exceptionally bright NIR‐II contrast agents through the synergistic marriage of chemical synthesis and ultrafast laser processing. Ag_2_Se QDs offer remarkable tissue penetration depth and spatiotemporal resolution, yet their photoluminescence (PL) efficiency is hindered by ions deficiency and crystal defects induced by the high mobility of Ag^+^ ions within Ag_2_Se crystals. Addressing this challenge, Wang and co‐workers et al. devised a tailored approach for controllable doping of Ag_2_Se QDs, achieved through Pb doping via cation exchange (CE), a feat unattainable via direct synthetic methods [58]. Termed Pb:Ag_2_Se QDs, these doped QDs exhibit novel optical properties, notably a 4.2‐fold enhancement in PL intensity. Photoelectron spectroscopy confirms Pb's role as an n‐type dopant, furnishing additional carriers to fill traps within Ag_2_Se QDs. Furthermore, the versatility of this method is demonstrated by successfully converting Ag_2_Se QDs of various sizes into Pb:Ag_2_Se QDs, thereby yielding a broad range of intense NIR‐II PL emissions. The bright NIR‐II emission of Pb:Ag_2_Se QDs is further showcased in successful lymphatic system mapping. Tumor‐lymph node (LN) metastasis stands as the paramount prognostic determinant for tumor staging and therapeutic stratagem. However, concurrently visualizing metastasis and executing imaging‐guided lymph node surgery presents a formidable challenge. Addressing this hurdle, Zhu and co‐workers et al. introduced a multiplexed NIR‐II in vivo imaging system employing nonoverlapping NIR‐II probes with significantly reduced photon scattering and zero‐autofluorescence, facilitating the visualization of metastatic tumors and the resection of tumor‐invaded proximal LNs [59]. A bright and tumor‐targeting donor‐acceptor‐donor (D‐A‐D) dye, IR‐FD, was meticulously selected for primary/metastatic tumor imaging in the NIR‐IIa (1100–1300 nm) window. This optimized D‐A‐D dye exhibits substantially enhanced quantum yield compared to organic D‐A‐D fluorophores in aqueous solutions (≈6.0 %) and demonstrates commendable in vivo performance. Furthermore, ultrabright PbS/CdS core/shell QDs, adorned with a dense polymer coating, are harnessed to visualize cancer‐invaded sentinel LNs in the NIR‐IIb (>1500 nm) window. In comparison to the clinically employed indocyanine green, these QDs exhibit superior brightness and photostability (no discernible bleaching even after continuous laser irradiation for 5 h), necessitating only a picomolar dose for sentinel LNs detection (Figure 3D). The amalgamation of dual‐NIR‐II image‐guided surgery can be seamlessly executed under bright light, augmenting its convenience and allure for clinical application. Dai and co‐workers et al. present an innovative study demonstrating the use of biocompatible core–shell lead sulfide/cadmium sulfide QDs emitting at approximately 1880 nm, in conjunction with superconducting nanowire single‐photon detectors [60]. This combination enables single‐photon detection up to 2000 nm, thereby establishing a one‐photon excitation fluorescence imaging window in the 1700–2000 nm (NIR‐IIc) range with 1650 nm excitation—the longest one‐photon excitation and emission achieved for in vivo mouse imaging to date. Utilizing confocal fluorescence imaging in the NIR‐IIc range, the researchers achieved an impressive imaging depth of approximately 1100 µm through an intact mouse head. Moreover, they facilitated non‐invasive cellular‐resolution imaging in the inguinal lymph nodes of mice without the need for surgery. Their methodology enabled in vivo molecular imaging of high endothelial venules with diameters as small as approximately 6.6 µm, as well as the visualization of CD169+ macrophages and CD3+ T cells in the lymph nodes. This study marks a significant advancement, opening avenues for non‐invasive intravital imaging of immune trafficking in lymph nodes at the single‐cell/vessel‐level longitudinally.

#### 3.2.2 Magnetic Resonance Imaging

**FIGURE 4:**
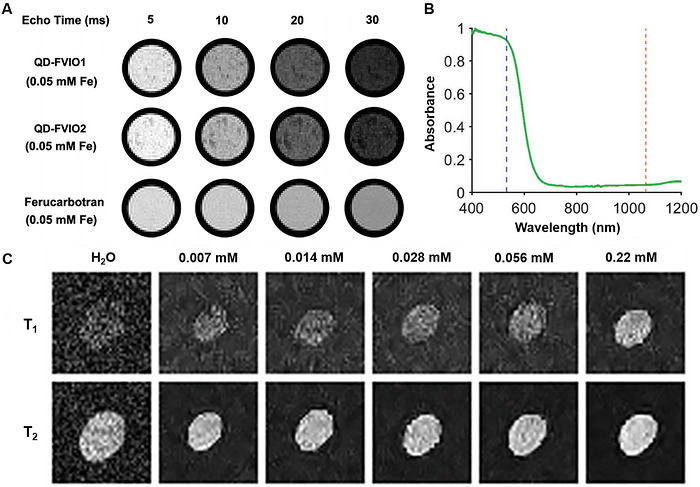
(A) In vitro T2*‐weighted MRI comparison between QD‐FVIOs in 2% agarose and commercial ferucarbotran in water [64]. (B) Absorption spectrum of CIS‐PDMS film fabricated via the dip‐coating method, emphasizing absorption peaks at 532 nm (blue dashed line) and 1064 nm (orange dashed line). Reproduced with permission [69]. Copyright 2021, Wiley‐VCH. (C) In vitro T1 and T2‐weighted MRI images with the increase of Mn(II) concentration. Reproduced with permission [68]. Copyright 2022, Springer Nature.

MRI plays a crucial role in clinical treatment owing to its high spatial resolution. Furthermore, MRI is renowned for being noninvasive and free of radiation [61, 62, 63]. Currently, paramagnetic relaxation signal agents such as Mn^2+^, Co^2+^, Fe^3+^, and Gd^3+^ are employed in MRI procedures. Additionally, MRI stands out as one of the most promising complementary techniques, providing valuable guidance for initial diagnoses and surgical planning of diseases. Its utilization significantly enhances detection accuracy and broadens the scope of practical applications. Fan et al. have successfully developed QD capped magnetite nanorings (NRs) with high luminescence and a magnetic vortex core, representing a novel class of magnetic‐fluorescent nanoprobe [64]. Through electrostatic interaction, cationic polyethylenimine (PEI) capped QDs have been firmly grafted onto negatively charged magnetite NRs modified with citric acid on the surface. The resulting biocompatible multicolor QD capped magnetite NRs exhibit a significantly stronger magnetic resonance (MR) T2* effect, with the r2* relaxivity and r2*/r1 ratio being 4 times and 110 times respectively larger than those of a commercial superparamagnetic iron oxide. Multiphoton fluorescence imaging and cell uptake of QD capped magnetite NRs are also demonstrated using MGH bladder cancer cells. Particularly noteworthy is the ability of these QD capped magnetite NRs to escape from endosomes and be released into the cytoplasm. The intensity values of QD‐FVIO MR images shown in Figure 4A have been adjusted for the T2* effects of agarose relative to water. Compared to ferucarbotran, QD‐FVIOs result in significantly greater signal reduction at the designated TE ranging from 10 to 30 ms. The findings from these exploratory experiments suggest that the cell‐penetrating QD capped magnetite NRs hold promise as an excellent dual‐modality nanoprobe for intracellular imaging and therapeutic applications. This work underscores the great potential of magnetic vortex core‐based multifunctional nanoparticles as high‐performance probes for biomedical applications. Naumov and co‐workers et al. have pioneered the development of novel biocompatible Mn‐NGQD and Gd‐NGQD structures for dual MRI and fluorescence imaging, employing a straightforward, cost‐effective, single‐step synthetic procedure [65]. These materials outperform many counterparts due to their superior biological and physical properties. Mn‐NGQDs and Gd‐NGQDs exhibit high biocompatibility even at concentrations exceeding 1 mg mL^−1^, attributed to the NGQD platform encapsulating metal ions. This addresses the critical toxicity concern associated with Gd‐based contrast agents, enabling safer imaging at elevated doses. Regarding their MRI contrast agent capabilities, Mn‐NGQDs demonstrate potential as dual‐mode T1/T2 contrast agents, while Gd‐NGQDs exhibit enhanced T1 characteristics with a markedly high longitudinal relaxivity rate, surpassing that of commercially available Magnevist (Gd‐DTPA). Their hydrophilicity and substantial surface‐to‐volume ratio facilitate interaction with water molecules, augmenting the MRI modality. Additionally, the functionally rich NGQD platform permits payload attachment, including targeting moieties, drugs, and genes. Furthermore, both Mn‐NGQDs and Gd‐NGQDs enable fluorescence image tracking intrinsic to the NGQD structure, validated in vitro by tracking their successful internalization into HEK‐293 cells. Consequently, the doped GQDs represent highly promising multifunctional platforms for future theranostics. Their dual MRI/fluorescence imaging modality opens avenues for numerous applications, such as fluorescence‐guided drug delivery tracking in vitro, MRI image tracking in vivo, and diagnostic and intraoperative imaging. Future investigations into fluorescence and MRI‐tracked drug delivery in vitro, in vivo, and ex vivo will corroborate the extensive theranostic potential of the Mn‐NGQDs/Gd‐NGQDs. Pong and co‐workers et al. introduced a multifunctional nanoplatform based on GQD‐CFO@SiO_2_ for monitoring drug delivery and fluorescence/MRI bimodal cellular imaging [66]. This nanoplatform was synthesized by conjugating GQDs onto the surface of magnetic CFO@SiO_2_ core/shell NPs, followed by covalent binding with FA. DOX was loaded onto the GQD surface via *π*–*π* stacking. The resulting DOX/GQD‐CFO@SiO_2_/FA facilitated drug delivery monitoring by detecting FRET signals. GQDs served as both drug carriers and donors in the FRET model, while DOX acted as the anticancer drug and acceptor. Controlled drug release was achieved via the FRET model. In vitro fluorescence imaging and T2‐weighted MRI bimodal cellular imaging demonstrated that GQD‐CFO@SiO_2_/FA could serve as a biomarker for targeted tumor tracking and drug delivery monitoring, leading to improved diagnosis accuracy. Additionally, DOX/GQD‐CFO@SiO_2_/FA exhibited significant cytotoxicity to HeLa cells, indicating enhanced therapeutic effectiveness. Consequently, the novel GQD‐CFO@SiO_2_/FA holds promise as a multifunctional nanoplatform for simultaneous cancer diagnosis and therapy through drug delivery monitoring and fluorescence/MRI bimodal cellular imaging. Yu and co‐workers et al. have successfully demonstrated a novel nanoprobe, TP‐CQDs@MnO_2_, for the dual‐mode fluorescence/MR imaging of intracellular H^+^ [67]. The MnO_2_ nanosheets not only quench the fluorescence signal of TP‐CQDs but also serve as carriers. Upon exposure to H^+^, the MnO_2_ nanosheets in TP‐CQDs@MnO_2_ are highly sensitive and undergo reduction to Mn^2+^, leading to the release of a significant amount of TP‐CQDs. The released TP‐CQDs and Mn^2+^ enable dual‐mode imaging, utilizing TP fluorescence and MR imaging, respectively. This dual‐mode imaging nanoprobe exhibits versatility in probing wide pH ranges (3.0–12.0) in vitro. Additionally, it effectively enables OP and TP fluorescence imaging, as well as MR imaging, across intracellular pH ranges (4.0–8.0). TP fluorescence imaging offers deeper tissue penetration (240.0 µm) and superior temporal and spatial resolution compared to OP fluorescence imaging of tissues. These remarkable properties position the dual‐mode imaging nanoprobe TP‐CQDs@MnO_2_ as a promising biological imaging tool for early diagnosis and clinical treatment of pH‐related diseases. Xu et al. introduced a TA‐Mn coordination polymer onto the surface of BCQD using a convenient in situ synthesis method, resulting in the successful fabrication of a novel MRI‐FL dual‐model imaging probe [68]. Rather than compromising the fluorescence of QDs, the incorporation of TA‐Mn coating causes a redshift in the emission wavelengths of CQDs, enhancing fluorescence imaging. Notably, BCQD@Mn(II) exhibits exceptional bright orange fluorescence properties with a quantum yield of 7.24%, alongside paramagnetic characteristics with r1 and r2 values of approximately 2.43 and 10.82 mM^−1^ S^−1^, respectively. Real‐time fluorescence imaging in vivo demonstrates the probe's facile entry into the bodies of mice, gradually attenuating over time. Ex vivo fluorescence imaging of organs reveals two metabolic pathways—liver and kidneys—indicating the probe's potential as an excellent dual‐modal imaging probe for enhanced MR imaging and fluorescence imaging. Figure 4C presents the T1 and T2 MRI images. While the brightness of both T1 and T2 images increases with the Mn(II) concentration, the enhancement of T1 images is significantly greater than that of T2 images. This indicates that the composite provides markedly enhanced MR signals in the in vitro imaging experiments.

#### 3.2.3 Photoacoustic Imaging

PAI, also referred to as optoacoustic imaging, is an emerging modality garnering growing interest in clinical research and translation, particularly in cancer management. This hybrid technique harnesses the benefits of optical excitation and acoustic detection, offering a complementary modality to conventional imaging technologies. Bodian et al. introduced the pioneering development of a quantum dot nanocomposite tailored for co‐registered laser‐generated ultrasound and photoacoustic imaging [69]. These nanocomposites, composed of CuInS_2_ QDs and medical‐grade polydimethylsiloxane (CIS‐PDMS), are applied onto miniature optical fibers' distal ends. The resulting films exhibit wavelength‐selective optical properties, demonstrating high optical absorption (>90%) at 532 nm for ultrasound generation and low absorption (<5%) at near‐infrared wavelengths beyond 700 nm (Figure 4B). When subjected to pulsed laser irradiation, the CIS‐PDMS films produce ultrasound with pressures surpassing 3.5 MPa and a corresponding bandwidth of 18 MHz. An ultrasound transducer is crafted by coupling the coated optical fiber with a Fabry‐Pérot (FP) fiber optic sensor. Leveraging the film's wavelength‐selective characteristics, co‐registered all‐optical ultrasound and photoacoustic imaging of an ink‐filled tube phantom is achieved. Chen and co‐workers et al. outlined a straightforward top‐down approach for synthesizing Bi_2_O_2_Se QDs in solution [70]. These QDs, measuring 3.8 nm in size and 1.9 nm in thickness, demonstrate a notable photothermal conversion coefficient of 35.7% and excellent photothermal stability. Both in vitro and in vivo evaluations highlight the exceptional photoacoustic (PA) performance and photothermal therapy (PTT) efficiency of Bi_2_O_2_Se QDs. Upon systemic administration, these QDs passively accumulate in tumors, facilitating comprehensive PA imaging of the entire tumor and enabling imaging‐guided PTT without noticeable toxicity. Furthermore, the degradable nature of Bi_2_O_2_Se QDs in aqueous media ensures adequate stability during in vivo circulation for therapeutic efficacy, followed by harmless discharge from the body.

Overall, QDs provide significant advantages in bioimaging due to their high brightness, resistance to photobleaching, and tunable emission in the NIR‐II window. These features enable deep‐tissue imaging and real‐time monitoring. Nevertheless, issues such as long‐term retention, potential toxicity, and unclear metabolic pathways continue to hinder their clinical translation.

### 3.3 Cancer Therapy

Cancer therapy is an ever‐evolving field, constantly seeking more effective and targeted treatments to combat the complex nature of cancer. QDs have emerged as innovative agents in this domain, offering unique photophysical properties that are harnessed for both therapeutic and diagnostic purposes in oncology. These nanoscale semiconductor particles exhibit distinct fluorescence, photostability, and tunable emission spectra, making them highly effective in various therapeutic and diagnostic applications.

**FIGURE 5:**
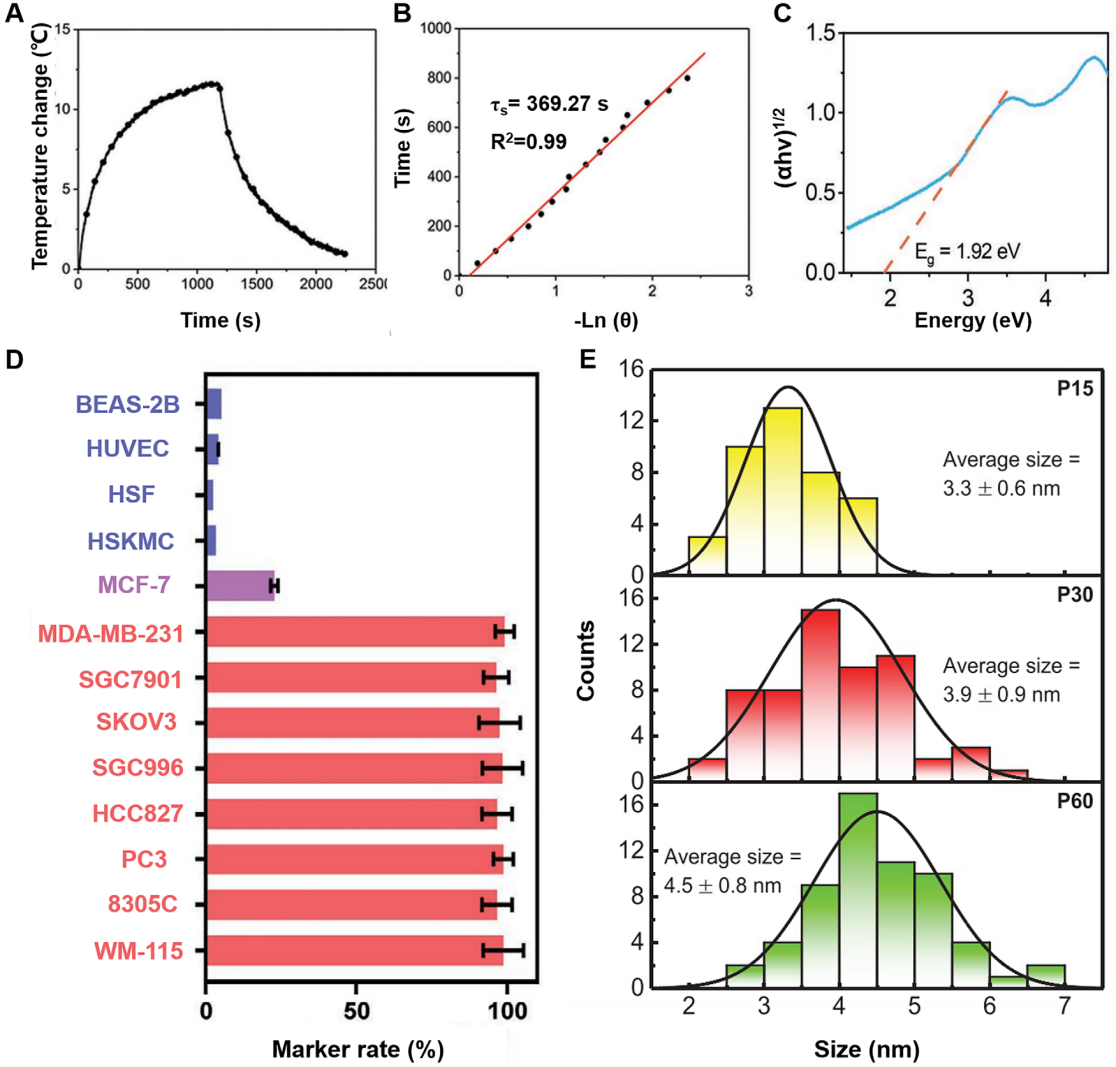
(A) Photothermal performance assessment of NCD solutions under 660 nm LED irradiation for 1200 s, followed by cessation of LED irradiation and natural cooling of the solution. (B) Linear time data plotted against ‐lnθ, derived from the cooling period depicted in Figure 5A. Reproduced with permission [72]. Copyright 2021, Wiley‐VCH. (C) The bandgap of the NCDs/MSN is obtained using the Tauc curve. Reproduced with permission [75]. Copyright 2023, Wiley‐VCH. (D) Binding rates comparison for different cell types of FA‐N‐GQDs. Reproduced with permission [76]. Copyright 2021, Wiley‐VCH. (E) Size distribution histograms of the P15 (top), P30 (middle), and P60 (bottom) samples, fitted with standard normal distribution curves. Reproduced with permission [77]. Copyright 2023, Wiley‐VCH.

Photodynamic therapy (PDT) emerges as a promising therapeutic approach against cancer due to its high spatiotemporal selectivity, utilizing photosensitizers (PSs) that activate only upon light irradiation, limiting toxicity. However, repetitive administrations required for optimal effectiveness often lead to severe side effects due to PS overdose. Addressing this challenge, Huang and co‐workers et al. introduced acidity‐activated graphene QDs‐based nanotransformers (GQD NT) as PS carriers for prolonged tumor imaging and repeated PDT [71]. Guided by the Arg‐Gly‐Asp peptide, GQD NT actively targets tumor tissues, expanding within the acidic tumor microenvironment, ensuring prolonged retention. Subsequently, GQD NT fragments into smaller pieces to enhance tumor penetration. Upon laser irradiation, GQD NT induces mild hyperthermia, augmenting cell membrane permeability and facilitating PS uptake. Remarkably, GQD NT not only illuminates fluorescence/magnetic resonance signals but also enables efficient repeated PDT. Notably, the total PS dose is reduced to 3.5 µmol kg^−1^, significantly lower than reported works. This study presents a smart vehicle that enhances PS accumulation, retention, and release in tumors through programmed deformation, overcoming the challenge of PS overdose in repeated PDT. The allure of injectable hydrogels capable of hosting a wide array of phototherapy agents, ensuring prolonged retention at tumor sites, has sparked considerable interest in concurrent photothermal and photodynamic cancer therapies. Yet, integrating these agents into hydrogels invariably necessitates intricate modifications. Furthermore, these agents often grapple with low efficacy and operate at wavelengths beyond the near‐infrared range. Zhu and co‐workers et al. have introduced a groundbreaking approach for crafting injectable hydrogels that enable simultaneous photothermal and photodynamic therapy [72]. They achieve this through a Schiff‐base reaction between amido‐modified carbon dots (NCDs) and aldehyde‐modified cellulose nanocrystals. The NCDs serve as both phototherapy agents and crosslinkers, facilitating hydrogel formation. Remarkably, the NCDs exhibit an exceptional photothermal conversion efficiency of 77.6% (Figure 5A,B), ranking among the highest for photothermal agents. Additionally, they boast a high singlet quantum yield of 0.37 under singular 660 nm light‐emitting diode irradiation. In vitro and in vivo animal experiments confirm the hydrogels’ non‐toxic nature and their effective inhibition of tumor growth. Thus, the direct integration of phototherapy agents into the hydrogel matrix not only presents novel avenues for injectable hydrogel fabrication but also charts a promising course for advanced tumor treatment strategies. Liu and co‐workers et al. developed a versatile nanoprobe (H‐MnO_2_/DOX/BPQDs) for dual‐modality cancer imaging and synergistic chemo‐phototherapy [73]. The process involved decorating hollow mesoporous MnO_2_ (H‐MnO_2_) nanoparticles with a cationic polymer poly (allylamine hydrochloride) (PAH) and an anionic polymer poly (acrylic acid) (PAA). Subsequently, H‐MnO_2_‐PAH‐PAA was covalently linked with BPQDs‐PEG‐NH_2_ through a carbodiimide cross‐linking reaction and loaded with the anti‐cancer drug DOX, resulting in the final nanoprobe H‐MnO_2_/DOX/BPQDs. Within the tumor microenvironment, H‐MnO_2_/DOX/BPQDs degraded to release encapsulated DOX and BPQDs. DOX functioned as both a chemotherapy and fluorescence imaging agent, while BPQDs conferred PDT and PTT abilities under dual laser irradiation at 630 and 808 nm. Additionally, H‐MnO_2_ provided contrast for MRI and facilitated the conversion of endogenous H_2_O_2_ to oxygen, thereby alleviating tumor hypoxia and enhancing PDT efficacy. Both in vitro and in vivo studies confirmed that the designed nanoprobe exhibited dual‐modality MRI/FL imaging and synergistic chemotherapy/PDT/PTT, ultimately improving the precision of cancer diagnosis and therapeutic outcomes. Gu and co‐workers et al. developed a sophisticated nanoplatform for targeted cancer therapy by harnessing the synergistic effects of various components [74]. Their approach involves hierarchically mesoporous metal–organic frameworks (HMMOFs) as the base, integrating black phosphorus QDs (BPQDs) and meso‐tetra (4‐carboxyphenyl) porphine (TCPP). The HMMOFs possess uniform large mesopores that facilitate the selective screening and immobilization of ultra‐small and monodispersed BPQDs. Meanwhile, TCPP located in microporous domains of HMMOFs can efficiently produce singlet oxygen (^1^O_2_), serving both as a photosensitizer for photodynamic therapy (PDT) and triggering the release of phosphate anions (PAs) from BPQDs in adjacent mesoporous domains. This dual action results in a synergistic effect termed as photo‐controlled synergistic interventional therapy (IIT). This nanoplatform, termed BP@HMUiO‐66‐TCPP, demonstrates excellent biocompatibility and biodegradability while exhibiting enhanced therapeutic effects. In murine models treated with BP@HMUiO‐66‐TCPP, the tumor inhibition rate reached approximately 98.24% after 14 days of treatment, significantly higher than the control group. Additionally, the tumor volumes in the synergetic group were only 19.6% of those in the PDT‐alone treated group. This study introduces a novel concept of exogenous photo‐controlled synergistic therapeutics, which holds promise for improving the efficacy of interventional cancer treatments. Hu and co‐workers et al. present a significant advancement in photodynamic therapy (PDT), particularly focusing on type I PDT, which offers reduced dependency on oxygen and addresses the challenge of hypoxia‐induced low efficacy against solid tumors [75]. Traditional metal‐based agents used in PDT pose long‐term biosafety concerns. To overcome this limitation, the researchers developed a metal‐free type I photosensitizer, N‐doped carbon dots/mesoporous silica nanoparticles (NCDs/MSN), with peroxidase (POD)‐like activity for synergistic PDT and enzyme‐based treatment on a gram scale. Their approach involves a facile one‐pot strategy, wherein a carbon source and silica precursor are mixed with the assistance of a template. The resulting nanohybrid, with a size of approximately 40 nm, benefits from the narrow bandgap (1.92 eV, Figure 5C) and excellent charge separation capacity of NCDs/MSN. Under 640 nm light irradiation, the excited electrons in the conduction band effectively generate O_2_^•−^ via a one‐electron transfer process, even under hypoxic conditions, inducing apoptosis in tumor cells. Additionally, the photoinduced O_2_^•−^ can further transform into the more toxic •OH through a two‐electron reduction. Moreover, the POD‐like activity of NCDs/MSN catalyzes the conversion of endogenous H_2_O_2_ to •OH in the tumor microenvironment, synergistically ablating 4T1 tumor cells. This study not only provides a mass production method for synthesizing a novel metal‐free type I photosensitizer with enzyme‐mimic activity but also demonstrates its promising clinical translation prospects for synergistic treatment of hypoxic tumors.

The advent of sonodynamic therapy (SDT) offers a promising avenue in tumor treatment. Its appeal lies in its safety, tissue penetration depth, and cost‐effectiveness. Unlike photodynamic therapy, which grapples with limited in vivo penetration (1–3 cm), SDT stands out for its controllable tissue penetration depth through ultrasound (US), rendering it suitable for clinical use. The mechanism behind SDT's efficacy involves irreversible tumor cell damage induced by heat generation, cavitation, and sonochemical processes triggered by US irradiation. Sonosensitizers, essential in this process, undergo sonochemical reactions upon US activation, resulting in the generation of reactive oxygen species (ROS) akin to those produced in photodynamic therapy. These ROS, including singlet oxygen (^1^O_2_), hydroxyl radical (•OH), and superoxide radical (O_2_^−^), play a pivotal role in enhancing the tumor cell‐killing capability of the US treatment. In this context, the sonochemical process assumes precedence over the heat effect of US irradiation in inflicting damage on tumors. Consequently, the efficacy of sonosensitization efficacy as a crucial metric in evaluating the performance of sonosensitizers in SDT. Inspired by the promising therapeutic potential of SDT and recognizing the catalytic capabilities of GQDs, Shi and co‐workers et al. marks the first successful demonstration of the exceptional SDT efficiency of nitrogen‐doped GQDs (N‐GQDs), attributed to their unique pyrrole N and pyridine N structures [76]. N‐GQDs exhibit a remarkable three to fivefold increase in singlet oxygen (^1^O_2_) generation efficiency compared to traditional sonosensitizers with porphyrin structures or metallic oxides. Through tumor‐targeting modifications, FA‐N‐GQDs have been identified as highly effective tumor‐targeted sonosensitizers, boasting a remarkable marker rate exceeding 96% (Figure 5D) for tumor cells and exceptional ROS generation efficiency. Combining FA‐N‐GQDs with ultrasound (US) treatment results in profound cancer cell eradication, with tumor inhibition rates surpassing 90%. These findings, validated through comprehensive in vitro and in vivo studies, offer profound insights into carbon nanostructure‐based SDT mechanisms, paving the way for a novel and efficient approach to cancer therapy.

One of the primary hurdles in achieving low‐cost, straightforward, and efficacious cancer treatments lies in the scarcity of intelligent anticancer drug delivery materials possessing site‐specific and microenvironment‐responsive attributes. Rao and co‐workers et al. present a breakthrough in this domain with their report on the creation of plasma‐engineered smart drug nanocarriers (SDNCs) comprising chitosan and NGQDs, designed for pH‐responsive drug delivery [77]. Through tailored microplasma processing, they achieve a highly cross‐linked SDNC, with a mere 4.5% (Figure 5E) N‐GQDs ratio, demonstrating a remarkable toughness increase of up to threefold compared to the control chitosan group. This advancement circumvents the necessity for high temperatures and toxic chemical cross‐linking agents commonly employed in conventional methods. The SDNCs exhibit heightened loading capacity for doxorubicin (DOX) facilitated by *π*–*π* interactions, along with stable solid‐state photoluminescence crucial for monitoring DOX loading and release through the Förster resonance energy transfer (FRET) mechanism. Furthermore, DOX‐loaded SDNCs demonstrate potent anticancer effects against cancer cells at minimal concentrations during cytotoxicity tests. Cellular uptake studies affirm successful internalization of DOX‐loaded SDNCs into the nucleus following a 12 h incubation period. This study heralds a new era in the development of smart, eco‐friendly, and biocompatible nanographene hydrogels poised for next‐generation biomedical applications.

Tumor metastasis accounts for nearly 90% of failures in cancer treatment and stands as the primary contributor to cancer‐related deaths, largely due to inadequate vascularization. In their study, Gu and co‐workers et al. pioneered the development of a sub‐50 nm hybrid theranostic nanoplatform using a template supramolecular approach [78]. This innovative platform enables active targeting and deep penetration of both primary tumors and metastatic lesions characterized by deficient vascular structures. The design involves the integration of QDs as a template, coupled with lipoic acid (LA)‐functionalized dendrimers for the covalent loading of doxorubicin (DOX). Additionally, Arg‐Gly‐Asp (RGD) tripeptide‐functionalized polyethylene glycol (PEG) is incorporated to prolong blood circulation and selectively target cancer cells. Upon internalization into tumor cells, the nanohybrid releases DOX within acidic lysosomes, facilitating its translocation into nuclei to induce cell cycle arrest at the G2/M phase. This mechanism yields a significant therapeutic effect on both primary tumors and distant metastases, as demonstrated in a 4T1 xenograft tumor model. Furthermore, the intrinsic fluorescence of QDs within the nanohybrid enables real‐time monitoring of therapeutic responses in both primary and metastatic tumors. This approach underscores a straightforward yet effective strategy for constructing a hybrid nanoplatform endowed with multifunctionality, thereby offering promising prospects for the inhibition of both primary and metastatic cancers.

CRISPR‐Cas9, a programmable gene‐editing tool with promising potential for cancer gene therapy, holds immense promise. Lee et al. harnessed this potential by delivering CRISPR ribonucleic protein (RNP) non‐covalently, using cationic glucosamine/PEI‐derived GQDs (PEI‐GQDs) [79]. These innovative delivery vehicles overcome physiological barriers and facilitate the tracking of genes of interest. The PEI‐GQD/RNP complex targets the tumor protein 53 (TP53) gene mutation, prevalent in approximately 50% of cancers, inducing double‐stranded breaks both in solution and within prostate cancer (PC‐3) cells. Restoring this cancer “suicide” gene initiates cellular repair pathways, triggering cancer cell apoptosis. Notably, simultaneous delivery of CRISPR RNP and a gene repair template via PEI‐GQDs yields a therapeutic outcome, resulting in 40% apoptotic cancer cell death, while sparing non‐cancerous (HeK293) cells. Tracking the translocation of the PEI‐GQD/RNP complex into PC‐3 cell cytoplasm is made possible through GQD intrinsic fluorescence, while the successful detachment of the gene editing tool upon internalization is evidenced by the detection of enhanced green fluorescent protein (EGFP)‐tagged RNP in the cell nucleus. This pioneering use of GQDs as non‐viral delivery and imaging agents for CRISPR‐Cas9 RNP sets the stage for image‐guided cancer‐specific gene therapy, offering a promising avenue for precision medicine in oncology.

### 3.4 Signal Transduction Mechanisms in QD‐Based Biomedical Systems

QDs enable a variety of signal transduction mechanisms that convert molecular interactions into detectable signals, forming the basis of their biomedical applications. Across the studies reviewed here, several recurring mechanisms can be identified. First, FRET is widely used in sensing and drug‐delivery systems, where distance‐dependent coupling between QDs and dyes, drugs, or biomolecular acceptors enables fluorescence modulation with high sensitivity [80]. Second, photoacoustic signal generation arises when absorbed optical energy is converted into transient thermoelastic expansion, allowing QD‐containing systems with strong optical absorption to function as contrast agents for deep tissue imaging [81]. Third, photothermal conversion is central to many therapeutic platforms, in which absorbed light is dissipated as heat to induce local hyperthermia, enhance membrane permeability, or synergize with drug release and photodynamic effects [82]. Fourth, redox‐responsive switching enables QD probes to respond dynamically to disease‐relevant microenvironments, such as oxidative stress, acidic conditions, or metal‐ion redox cycling, thereby improving contrast specificity and reducing background signal [83]. Fifth, enzyme‐mimetic catalysis provides an additional functional layer, particularly in catalytic therapy and biosensing, where QD‐based nanostructures can promote ROS generation or substrate conversion in a manner analogous to peroxidase or oxidase‐like systems [84]. Collectively, these mechanisms illustrate that the biomedical value of QDs lies not only in tunable emission, but also in their ability to integrate optical readout, chemical responsiveness, and therapeutic activation within a single nanoscale platform.

Overall, QDs enable multifunctional integration of diagnosis and therapy, offering a promising platform for precision medicine. However, their therapeutic performance is closely linked to surface chemistry, targeting efficiency, and photophysical stability, which also introduce complexity in safety evaluation and clinical translation.

## 4 Challenges and Optimization Strategies for QDs in Biomedical Applications

The biomedical applications of QDs are still hindered by multiple interrelated barriers spanning material design, biological properties, and scalable manufacturing, as detailed below.

### 4.1 Toxicity

Nanocatalysts leveraging Fenton or Fenton‐like reactions to enhance intracellular oxidative stress have emerged as a leading edge in tumor‐specific therapy. However, the translation of these advancements encounters significant hurdles, such as low catalytic efficiency, inadequate biocompatibility, and potential toxicities. Liu and co‐workers et al. propose a promising solution for cancer treatment by harnessing a Ti‐based material renowned for its exceptional biocompatibility [85]. Through a self‐designed microexplosion method, they successfully synthesize nonoxidized MXene‐Ti_3_C_2_T_x_ QDs (NMQDs‐Ti_3_C_2_T_x_). Remarkably, these QDs exhibit a marked inhibitory effect on cancer cells while maintaining excellent compatibility with normal cells. Moreover, in xenograft tumor models, NMQDs‐Ti_3_C_2_T_x_ achieve an impressive suppression rate of 91.9% without compromising surrounding healthy tissues. Mechanistically, the Ti^3+^ species within NMQDs‐Ti_3_C_2_T_x_ react with H_2_O_2_ in the tumor microenvironment, producing a surplus of toxic hydroxyl radicals that enhance tumor microvascular permeability and collaboratively eliminate cancer cells. The promising potential of Ti‐based Fenton‐like reaction catalytic therapy is poised for expansion, driven by the acknowledged biocompatibility of Ti‐based materials and the demonstrated ability of Ti^3+^ to selectively target and eradicate cancer cells.

### 4.2 Biocompatibility

Quantum metrology stands at the forefront of precision measurement, offering unparalleled accuracy. In the field of life sciences, diamond‐based quantum sensing has introduced a new class of biophysical sensors and diagnostic devices. These advancements hold promise as a platform for cancer screening and ultrasensitive immunoassays. However, the broader application of nanoscale NMR spectroscopy in life sciences has faced biocompatibility challenges due to the intricate task of interfacing highly sensitive quantum bit (qubit) sensors with biological targets. Addressing this challenge, Maurer and co‐workers et al. present a groundbreaking approach that amalgamates quantum engineering with single‐molecule biophysics [86]. Their method involves immobilizing individual proteins and DNA molecules onto the surface of a bulk diamond crystal hosting coherent nitrogen‐vacancy qubit sensors. Notably, their thin (sub‐5 nm) functionalization architecture offers precise control over biomolecule adsorption density, yielding near‐surface qubit coherence approaching 100 µs. Crucially, this developed architecture exhibits exceptional chemical stability under physiological conditions for over 5 days, rendering the technique compatible with a myriad of biophysical and biomedical applications. By seamlessly integrating highly sensitive quantum sensors with biological entities, Maurer et al.’s approach opens avenues for unprecedented precision and sensitivity in diagnostics and biophysical studies.

### 4.3 Low Accumulation

Hepatic ischemia‐reperfusion injury (HIRI) is a critical clinical challenge following liver surgery, particularly impacting the prognosis of patients with end‐stage liver disease. Reactive oxygen species (ROS) play a key driving role in the development of HIRI, directly contributing to liver dysfunction. Although selenium‐doped CQDs (Se‐CQDs) exhibit excellent redox‐responsive properties, enabling efficient neutralization of ROS and protection against oxidative cellular damage, their ability to achieve targeted accumulation in the liver remains relatively limited. To tackle this issue, Xia and co‐workers et al. incorporated Se‐CQDs into lecithin nanoparticles (Se‐LEC NPs) through self‐assembly driven mainly by noncovalent interactions [87]. Lecithin, as a crucial component, enhances the therapeutic efficacy of Se‐LEC NPs by its ROS‐reactive capability. Consequently, the Se‐LEC NPs demonstrate significant liver accumulation, efficiently scavenging ROS and curbing the release of inflammatory cytokines, thereby exhibiting promising therapeutic benefits against HIRI. This innovative approach may pave the way for designing self‐assembled Se‐CQDs NPs for treating not only HIRI but also other ROS‐related diseases.

### 4.4 In Vivo Activity, Biosafety, and Translational Challenges

Beyond intrinsic toxicity and biocompatibility, their clinical translation is still severely hampered by multifaceted dilemmas in in vivo metabolism, biosafety, immunogenicity, biodegradability, repeatability, and regulatory standardization. In terms of in vivo metabolism, most QDs are prone to non‐specific distribution and long‐term retention in the liver, spleen, and other organs, with low renal clearance efficiency and unknown metabolic pathways of heavy metal core components, leading to potential long‐term accumulation risks. For biosafety, the release of heavy metal ions from damaged inorganic cores triggers oxidative stress, organ toxicity, and genotoxicity, and the lack of long‐term and low‐dose exposure toxicity data further limits its clinical application. In the aspect of immunogenicity, surface modification ligands and nanoparticle itself can activate innate and adaptive immune responses, induce inflammatory factor release and specific antibody production, resulting in accelerated clearance and hypersensitivity reactions. Biodegradability is another critical obstacle: traditional inorganic QD cores are almost non‐biodegradable in vivo, while degradable alternative QDs suffer from compromised fluorescence performance and stability. Additionally, the batch‐to‐batch differences in the synthesis process, non‐uniform surface modification, and lack of unified characterization standards result in poor repeatability of experimental results, making it difficult to ensure product consistency. Laboratory‐scale protocols typically rely on precisely controlled conditions that cannot be directly replicated on industrial production lines, resulting in a clear disconnect between laboratory‐scale synthesis and large‐scale industrial manufacturing. Finally, the lack of unified clinical regulatory standards further impedes the translation of QDs. Current evaluation frameworks, largely adapted from conventional nanomaterials and pharmaceuticals, are insufficient to fully address the complexity of QDs systems. The establishment of standardized characterization methods, long‐term safety evaluation protocols, and dedicated regulatory guidelines will be critical for advancing QDs toward clinical applications.

As illustrated in Table 1, different classes of QDs each possess their own advantages, disadvantages, and applicable scenarios, and there is no universal optimization paradigm. Traditional Cd‐based QDs remain among the most optically mature systems, with high brightness, narrow emission, and well‐developed surface chemistry, but their clinical potential is restricted by heavy‐metal toxicity concerns. Cd‐free alternatives such as InP‐based QDs reduce this concern but still require careful control of surface defects and long‐term stability. Carbon dots and graphene quantum dots generally offer the most favorable biocompatibility profile and synthetic versatility, although they often face challenges in spectral precision, structural heterogeneity, and quantitative reproducibility. Perovskite QDs provide highly attractive optical properties but remain limited by intrinsic instability and, in many cases, Pb‐related safety concerns. Ag_2_X NIR‐II QDs are particularly promising for deep‐tissue imaging because they combine extended‐wavelength emission with relatively favorable biocompatibility, yet their long‐term fate, compositional stability, and large‐scale standardization still require further study. Customized translational design must be carried out according to specific biomedical demands. Future translational research on QDs should abandon the mindset of “prioritizing performance over translation,” and take material safety, controllable in vivo behavior, batch reproducibility, and manufacturing scalability as core design parameters, integrating them into the entire process from material synthesis to application development. Through the cross‐integration of multiple disciplines, we aim to advance QDs from technological innovation in the laboratory to truly clinically accessible, safe, and effective biomedical tools.

**TABLE 1:** Comparison of representative QDs.

Characteristic	Cd‐based QDs	Cd‐free QDs	Carbon/Graphene QDs	Perovskite QDs (PQDs)	Ag_2_X NIR‐II QDs (X = S/Se/Te)
Typical Emission Range	Visible to NIR‐I (400–900 nm)	Visible to NIR‐I (400–900 nm)	Visible to NIR‐I (400–900 nm)	Visible to NIR‐I (400–900 nm)	NIR‐II (900–1700 nm)
Core Optical Advantages	High brightness, narrow emission peak, high quantum yield	Low heavy metal toxicity, tunable optical performance	Excellent intrinsic biocompatibility, low synthesis cost, facile functionalization	Ultra‐high quantum yield, broad absorption spectrum, high light‐harvesting efficiency	Deep tissue penetration, low photon scattering, zero autofluorescence, tunable NIR‐II emission
Biocompatibility	Poor; Cd^2^^+^ leakage induces significant cytotoxicity and in vivo toxicity	Good; no cadmium‐related toxic risks, low ion leakage potential	Excellent; low toxic, good water dispersibility, low immunogenicity	Moderate; Pb‐containing systems pose ion leakage and chronic toxicity risks	Good; better biocompatibility than traditional heavy metal QDs, low in vivo cytotoxicity
Stability	Good; high photostability and colloidal stability, mature surface passivation	Moderate; prone to surface defects, easy to cause optical performance attenuation	Good; high chemical and photostability, stable in physiological media	Poor; susceptible to degradation by water, oxygen, and light, poor colloidal stability	Medium‐Good; core–shell engineering effectively improves photostability and structural stability
Main Biomedical Applications	Ultrasensitive biosensing, fluorescence imaging, photodynamic therapy (PDT)	Biosensing, targeted fluorescence imaging, stimulus‐responsive drug delivery	Biosensing, multimodal imaging, enzyme‐mimetic catalysis therapy, sonodynamic therapy (SDT)	High‐sensitivity biosensing, high‐resolution fluorescence imaging, photothermal conversion	Deep‐tissue in vivo imaging, intraoperative navigation, precision cancer therapy, NIR‐II fluorescence‐guided phototherapy
Key Challenges	Severe heavy metal toxicity limits clinical translation; potential long‐term in vivo retention	Difficult control of surface defects; insufficient long‐term stability and batch reproducibility	Low spectral precision; structural heterogeneity; limited brightness for deep‐tissue imaging	Intrinsic instability; Pb ion leakage risk; poor in vivo stability	Unclear long‐term metabolic fate and excretion pathway; difficult large‐scale standardized synthesis
Translational Potential	Low	Medium	High	Low‐Medium	High
Representative Optimization Strategies	Core–shell structure passivation; surface ligand engineering to reduce Cd^2^^+^ leakage	Surface defect passivation; high‐quality ligand modification to enhance stability	Doping modification; structural regulation to improve spectral precision and brightness	Encapsulation modification; component regulation to enhance environmental stability	Core–shell design; surface PEGylation to improve in vivo circulation and targeting ability

In summary, the clinical translation of QDs represents a systematic endeavor encompassing material science, biology, manufacturing, and regulatory compliance, with its core contradiction lying in the balance between performance optimization and translational feasibility. The physicochemical characteristics at the intrinsic material level form the foundation for QDs to exert biological functions, yet they may also give rise to core issues such as toxicity and poor stability. The complex in vivo biological microenvironment will further alter the actual behavior of QDs, leading to risks including reduced targeting efficiency and long‐term retention. Moreover, the disconnect between laboratory‐scale fabrication processes and large‐scale manufacturing, as well as regulatory requirements, stands as a critical barrier hindering the translation of QDs from the laboratory to clinical practice.

## 5 Summary and Perspectives

QDs, distinguished by their tunable emission, high brightness, and robust photostability, have positioned themselves at the forefront of nanotechnology, driving innovations in biosensing, bioimaging, and precision cancer therapy. This review has highlighted the significant progress in the synthesis, functionalization, and application of QDs, demonstrating their versatility and potential in enhancing diagnostic and therapeutic practices. In biosensing, QDs have shown promise in improving the detection of various biomarkers and infectious agents, providing tools that are more sensitive and specific than traditional methods. The ability of QDs to function as fluorescent probes in bioimaging has revolutionized techniques such as MRI, PAI, and fluorescence imaging, offering deeper tissue penetration and higher resolution, which are crucial for accurate diagnosis and monitoring of diseases. In the realm of cancer therapy, QDs have been instrumental in developing novel photodynamic therapy approaches that minimize side effects associated with traditional treatments. By facilitating targeted drug delivery and controlled release mechanisms, QDs enhance the efficacy of treatments, reducing the dosage requirements and mitigating associated toxicities.

Nevertheless, the clinical translation of QDs remains fraught with challenges. Current research efforts are primarily focused on two key dimensions: material optimization and targeted delivery. To address toxicity and biocompatibility issues, on one hand, developing biocompatible coatings to reduce immunogenicity; On the other hand, actively exploring the replacement of traditional heavy‐metal‐based QDs with non‐toxic alternatives such as cadmium‐free and carbon‐based QDs, thereby enhancing biosafety at the source. Meanwhile, to improve the accumulation and retention of QDs at lesion sites, various targeted delivery strategies are being designed—including the conjugation of specific ligands and the construction of smart responsive nanocarriers, aiming to enhance therapeutic efficacy while minimizing systemic exposure, thus advancing both the safety and effectiveness of QD‐based clinical applications.

In the long term, the development of QD technology will be characterized by multidimensional integration and interdisciplinary collaboration. Within the biomedical domain, QDs are becoming deeply integrated with conventional medical technologies such as biomedical imaging and minimally invasive surgery, gradually expanding into cutting‐edge applications including single‐cell tracking and intraoperative real‐time navigation, where they demonstrate exceptional spatiotemporal resolution and sensitivity. Beyond these established areas, the convergence of QDs with emerging technologies, including gene editing, photothermal and photodynamic therapy, and smart drug delivery systems, is giving rise to integrated intelligent theranostic platforms that combine diagnosis, therapy, and monitoring. It is worth emphasizing that this progress relies heavily on the synergistic efforts of multiple disciplines, such as nanotechnology, chemistry, biology, materials science, and clinical medicine. Interdisciplinary integration will serve as the core engine driving a qualitative leap in QDs technology.

Not only that, the journey from bench to bedside still requires overcoming a series of systemic challenges. As the functional design of QDs becomes increasingly sophisticated, key issues such as their in vivo metabolic behavior, long‐term toxicity, and environmental fate remain insufficiently evaluated. Therefore, the establishment of standardized and regulated safety assessment systems has become an urgent priority. At the same time, the lag in regulatory policies and the absence of ethical review mechanisms continue to impede clinical translation. Only by advancing technological innovation while proactively constructing regulatory frameworks and ethical guidelines can we ensure that QDs, as a cutting‐edge tool, serve the long‐term development of human health in a safe, controllable, and sustainable manner.

## Conflicts of Interest

The authors declare no conflicts of interest.

## Data Availability

Data sharing not applicable to this article as no datasets were generated or analyzed during the current study.
